# Risk factors for pathological upgrading in perimenopausal women with cervical intraepithelial neoplasia grade 2/3 following conization

**DOI:** 10.1097/MD.0000000000031368

**Published:** 2022-10-28

**Authors:** Mingyu Jia, Chong Lan, Jumin Niu, Yin Liang

**Affiliations:** a Graduate School, Dalian Medical University, Dalian, Liaoning, China; b Department of Gynecology, Shenyang Women and Children’s Hospital, Shenhe District, Shenyang, Liaoning, China.

**Keywords:** advanced cervical intraepithelial neoplasia, cervical cancer, conization, pathological upgrading

## Abstract

Postmenopausal women have a high risk for pathological upgrading in conization specimens due to pathological changes of the cervix. This study aimed to investigate the risk factors for pathological upgrading in conization specimens in Chinese women with cervical intraepithelial neoplasia grade 2/3 (Cervical intraepithelial neoplasia 2/3) ≥ 50 years of age. From January 2015 to December 2019, 443 CIN2/3 patients ≥ 50 years of age were retrospectively included and divided into the upgrade group (n = 47) and the non-upgrade group (n = 396) according to the presence or absence of pathological upgrading in the conization specimens. Multivariate logistic regression model was performed to analyze risk factors associated with pathological upgrading. The upgrade group was more likely to have gravidity < 2 times, postmenopausal period ≥ 5 years, higher incidences of endocervical glandular involvement (EGI) and human papillomavirus (HPV) 16/18 infection, as well as a lower incidence of cervical contactive bleeding and fewer cases undergoing endocervical curettage (all *P* < .05) than the non-upgrade group. Multivariate model showed that factors associated with pathological upgrading were postmenopausal period ≥ 5 years (OR = 2.55), EGI (OR = 17.71), endocervical curettage (OR = 0.33), and HPV type 16/18 (OR = 3.41) (all *P* < .05). The receiver operating characteristic analysis showed an area under curve of 0.782 (*P* < .001). Pathological upgrading in conization specimens is not uncommon in Chinese CIN2/3 patients ≥ 50 years of age. For those with high-risk factors of pathological upgrading (postmenopausal period ≥ 5 years, EGI, and HPV 16/18 infection), the follow-up interval can be appropriately shortened, and active intervention could be considered.

## 1. Introduction

Cervical cancer is the fourth most common cancer among women worldwide in 2020, with approximately 90% of the new cases and deaths occurring in low- and middle-income countries.^[1]^ Cervical intraepithelial neoplasia (CIN) is a premalignant squamous lesion of the uterine cervix,^[2]^ first described by Richart in 1967^[3]^ and subsequently by Crum in 1982,^[4]^ and characterized by abnormal growth of squamous cells on the surface of the cervix.^[5]^ CIN2 and CIN3 are high-grade lesions with a higher risk of progression to cervical cancer compared to CIN1.^[6]^ The current guidelines suggest that advanced CIN should be timely diagnosed and actively treated with surgery to prevent the disease from progressing to cervical cancer.^[7,8]^

The CIN is mainly diagnosed by colposcopic-directed cervical biopsy.^[9]^ Surgical treatment of CIN2/3 includes cold knife conization (CKC) and loop electrical excision procedure (LEEP), which can provide pathological findings of interstitial infiltration for further diagnosis.^[10]^ However, in clinical practice, a certain proportion of CIN patients encounter pathological upgrading in conization specimen as compared with colposcopic-directed cervical biopsy.^[11,12]^ The probability of pathological upgrading following cervical conization elevates with decreasing estrogen level, the receding of the squamocolumnar junction, and atrophic changes in the postmenopausal cervix.^[13]^

At present, studies on pathological upgrading in perimenopausal women are rare.^[14]^ Investigation of risk factors for postoperative pathological upgrading may help reduce the possibility of missed diagnosis rate. This study aimed to investigate the risk factors for pathological upgrading following conization in women with CIN2/3 ≥ 50 years of age.

## 2. Materials and methods

### 2.1. Study subjects

From January 2015 to December 2019, 443 CIN2/3 patients treated in Shenyang Women’s and Children’s Hospital were retrospectively included. The inclusion criteria were: patients were diagnosed with CIN2/3 by colposcopy cervical biopsy and received conization (CKC or LEEP) for the first time; age ≥ 50 years.

Exclusion criteria were: pregnant women; history of cervical ablation or surgical treatment, including microwave, cryotherapy, laser therapy, LEEP, CKC, total hysterectomy, subtotal hysterectomy, transcervical resection; combined with severe diseases; reproductive tract infections, obvious exogenous or ulcerative lesions on the surface of the cervix; combined with endometrial polyps, submucosal uterine fibroids that required hysteroscopic surgery; incomplete medical records.

Pathological upgrading was defined as histological results progression in ≥ 2 sites (histological results at colposcopy cervical biopsy vs histological results at conization, CIN2 progression to CIN3; CIN2/3 progression to cervical cancer). According to the pathological results at the conization specimen, the patients were divided into the upgrade group (there were ≥ 2 sites of pathological upgrading compared with the cervical biopsy results, n = 47), and the non-upgrade group (cervical intraepithelial neoplasia, inflammation or normal, n = 396). The histological results at conization compared with those of the cervical biopsy before the excisional treatment was shown in Table 1. This study was approved by the institutional review board of Shenyang Women and Children’s Hospital [no. 201917]. Written informed consent was obtained from the patient.

**Table 1:** Histological results at conization compared with those of the cervical biopsy before the excisional treatment.

Histological results	Cases number
**^a^Non-upgrade group (n = 396**)	
CIN2	217
CIN3	179
**^b^Upgrade group (n = 47**)	
CIN2 → CIN3	35
CIN2 → cervical cancer	5
CIN3 → cervical cancer	7

CIN = cervical intraepithelial neoplasia.

a In the Non-upgrade group, the histological result was the same at the cervical biopsy before the excisional treatment and conization.

b In the upgrade group, the histological results progression occurred between the cervical biopsy before the excisional treatment and the conization specimen.

### 2.2. Data collection

Patients’ demographic and clinical data were retrospectively collected from the medical record and compared between the 2 groups, including age, gravidity, body mass index, postmenopausal period, endocervical glandular involvement (EGI), endocervical curettage (ECC), human papillomavirus (HPV) type 16/18, and cervical contactive bleeding.

### 2.3. Statistical analysis

Continuous data were presented as mean ± standard deviation while categorical data were reported as number and percentage (%). For comparisons of means between groups, the student’s independent *t* test or Mann–Whitney *U* test was used depending on the assumption of normality. Categorical data were tested using the Chi-square test or Fisher’s exact test (if expected value ≤ 5 was observed).

Univariate and multivariate logistic regression models were used to investigate the factors associated with pathological upgrading. The independent variables which were significant in univariate results were included in a multivariate model, and the variables which were significant in the multivariate model were recognized as independent factors associated with pathological upgrading. The estimated odds ratio (OR) was reported. The final risk or protective factors were used to build a multivariate logistic model and to estimate the probability of pathological upgrading of each patient. The estimated probabilities would be examined as independent continuous variables in a receiver operating characteristic (ROC) analysis and the area under curve was reported.

A *P* < .05 was considered significant, 2-tailed. All above analyses were performed using IBM SPSS Version 25 (SPSS Statistics V25, IBM Corporation, Somers, New York). The nomogram was built by the risk and protective factors through statistical software R (version 3.5.2) and package “rms”.

## 3. Results

### 3.1. Patient’s clinical characteristics

A total of 443 patients (mean age = 57.47 ± 4.98 years) with CIN 2 to 3 were included and divided into upgrade group (n = 47, 10.61%) and non-upgrade group (n = 396, 89.39%). As shown in Table 2, patients’ age and body mass index were not significantly different between the 2 groups (both *P* > .05).

**Table 2:** Patients’ demographic and clinical characteristics.

Parameters	Non-upgrade (n = 396)	Upgrade (n = 47)	All (n = 443)	P
Age, year	57.33 ± 5.00	58.62 ± 4.70	57.47 ± 4.98	0.094
Gravidity				0.013
<2	169 (42.68%)	29 (61.70%)	198 (44.70%)	
≥2	227 (57.32%)	18 (38.30%)	245 (55.30%)	
BMI, kg/m^2^	24.21 ± 3.18	24.02 ± 2.74	24.19 ± 3.14	0.698
Postmenopausal period ≥ 5 years				0.011
No	141 (35.61%)	8 (17.02%)	149 (33.63%)	
Yes	255 (64.39%)	39 (82.98%)	294 (66.37%)	
EGI				<0.001
No	119 (30.05%)	2 (4.26%)	121 (27.31%)	
Yes	277 (69.95%)	45 (95.74%)	322 (72.69%)	
ECC				0.031
No	204 (51.52%)	32 (68.09%)	236 (53.27%)	
Yes	192 (48.48%)	15 (31.91%)	207 (46.73%)	
HPV type 16/18				0.001
No	219 (55.30%)	14 (29.79%)	233 (52.60%)	
Yes	177 (44.70%)	33 (70.21%)	210 (47.40%)	
Cervical contactive bleeding				0.013
No	177 (44.70%)	30 (63.83%)	207 (46.73%)	
Yes	219 (55.30%)	17 (36.17%)	236 (53.27%)	

BMI = body mass index, ECC = endocervical curettage, EGI = endocervical glandular involvement, HPV = human papillomavirus.

The upgrade group was more likely to have gravidity < 2 times (61.70% vs 42.68%), postmenopausal period ≥ 5 years (82.98% vs 64.39%), higher incidences of endocervical glandular involvement (EGI, 95.74% vs 69.95%), and HPV 16/18 infection (70.21% vs 44.70%), as well as a lower incidence of cervical contactive bleeding (36.17% vs 55.30%) and fewer cases undergoing endocervical curettage (ECC, 31.91% vs 48.48%) (all *P* < .05) as compared with the non-upgrade group.

### 3.2. Risk factors associated with pathological upgrading

To investigate the independent factors associated with pathological upgrading, univariate and multivariate logistic regression models were performed. As shown in Table 3, multivariate model showed that associated factors were postmenopausal period ≥ 5 years (OR = 2.55, 95% confidence interval (CI) = 1.11 to 5.86), EGI (OR = 17.71, 95% CI = 4.05 to 77.51), ECC (OR = 0.33, 95% CI = 0.16 to 0.66), and HPV type 16/18 (OR = 3.41, 95% CI = 1.69 to 6.87) (all *P* < .05). These results indicated that patients with postmenopausal period ≥ 5 years, EGI, and HPV type 16/18 were more likely to have pathological upgrading, whereas patients receiving ECC were less likely to have pathological upgrading.

**Table 3:** The logistic regression results of independent variables associated with pathological upgrading.

	Univariate		Multivariate		Final model	
Parameters	OR (95% CI)	P	OR (95% CI)	P	OR (95% CI)	P
Age, year	1.05 (0.99 to 1.11)	0.095				
Gravidity						
<2						
≥2	0.46 (0.25 to 0.86)	0.015	0.73 (0.10 to 5.18)	0.754		
BMI, kg/m^2^	0.98 (0.89 to 1.08)	0.697				
Postmenopausal period ≥ 5 years						
No						
Yes	2.70 (1.23 to 5.93)	0.014	2.55 (1.11 to 5.86)	0.027	3.00 (1.30 to 6.96)	0.010
EGI						
No						
Yes	9.67 (2.31 to 40.50)	0.002	17.71 (4.05 to 77.51)	<0.001	13.60 (3.16 to 58.56)	<0.001
ECC						
No						
Yes	0.50 (0.26 to 0.95)	0.034	0.33 (0.16 to 0.66)	0.002	0.35 (0.18 to 0.70)	0.003
HPV type 16/18						
No						
Yes	2.92 (1.51 to 5.62)	0.001	3.41 (1.69 to 6.87)	<0.001	3.76 (1.88 to 7.51)	<0.001
Cervical contactive bleeding						
No						
Yes	0.46 (0.24 to 0.86)	0.015	0.49 (0.07 to 3.51)	0.480		

BMI = body mass index, CI = confidence interval, ECC = endocervical curettage, EGI = endocervical glandular involvement, HPV = human papillomavirus, OR = odds ratio.

### 3.3. ROC analysis and nomogram

**Figure 1.**
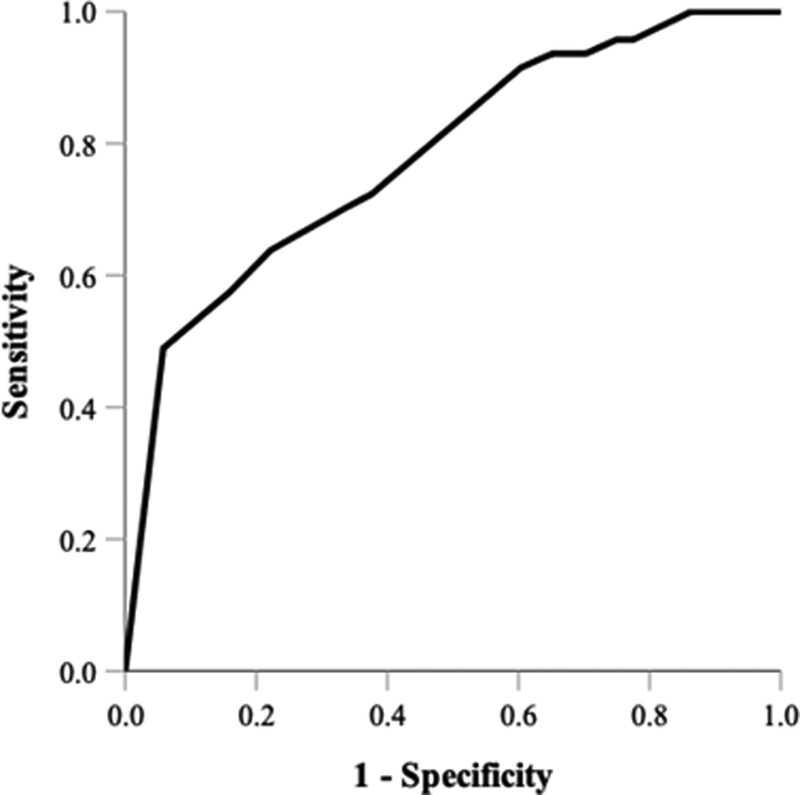
The ROC curve of the probabilities was estimated by factors associated with pathological upgrading. (AUC = 0.782, 95% CI = 0.709 to 0.854; *P* < .001). AUC = area under curve, CI = confidence interval, ROC = receiver operating characteristic.

Based on the multivariate logistic regression model, the probabilities of pathological upgrading were estimated in the ROC analysis (Fig. 1). The ROC analysis showed an area under curve of 0.782 (95% CI = 0.709 to 0.854; *P* < .001), suggesting fair predictability.

**Figure 2.**
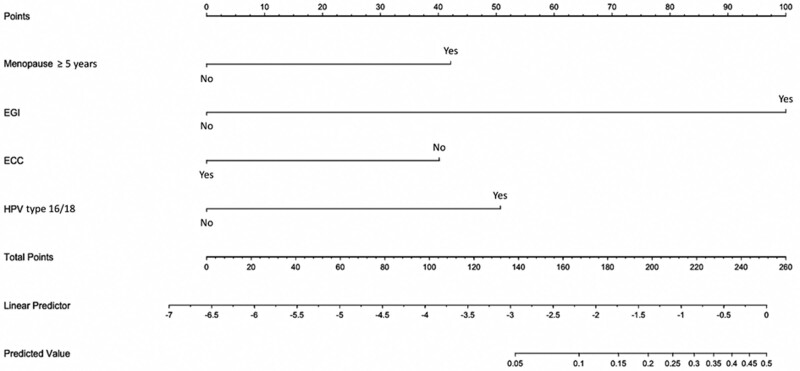
The nomogram of pathological upgrading which was established by associated factors, including postmenopausal period ≥ 5 years, EGI, ECC, and HPV type 16/18. EGI = endocervical glandular involvement, ECC = undergoing endocervical curettage, HPV = human papillomavirus.

A nomogram was also established by this model (Fig. 2).

## 4. Discussion

In perimenopausal women, the decline of estrogen levels causes a series of physiological changes: the levels of cervical squamous epithelial cells, the stromal blood vessels, and glycogen are reduced so that the cervix poorly responds to acetic acid, and the cervical iodine staining shows uneven yellow. Meanwhile, the columnar epithelium shows atrophy and loses its typical villus-like structure. The volume of individual cells decreases, and the cervix appears pale and atrophy. The whole cervix becomes narrower, its depth and length increase, and the squamous epithelium of the cervix and vagina moves inward. These physiological changes reduce the accuracy of colposcopy evaluation and colposcopic biopsy and cytology.^[15–19]^ Thereby, suspicious lesions are difficult to identify or even missed, resulting in pathological upgrading in conization specimens. Supporting this notion, Costa et al^[20]^ analyzed the pathological results of 739 high-grade squamous intraepithelial lesions patients following conization and found that patients over 50 years of age had an 11-fold higher risk of missed diagnosis of cervical cancer by colposcopy as compared with those under 30 years old. In Chinese women, the mean age at menopause is around 49 to 50 years old,^[21–23]^ and nearly 80% of them have menopause at the age of 46 to 54 years old.^[24]^ Therefore, in this study, we investigated the risk factors for pathological upgrading in Chinese women with CIN2/3 ≥ 50 years of age.

High-risk HPV infection is an important factor for the development of CIN and cervical cancer.^[25,26]^ More than 90% of high-grade CIN or cervical cancer cases are associated with high-risk HPV infection,^[27]^ with HPV 16/18 infection accounting for approximately 70% of cervical cancers.^[28]^ In this study, of 210 cases (47.40%) with HPV16/18 infection, 33 cases (15.7%) had postoperative pathological upgrading. Among the 233 cases (52.60%) with other types of high-risk HPV infection, 14 cases (6.0%) had postoperative pathological upgrading. The pathological upgrading rate was significantly elevated in patients with HPV16/18 infection than those with other types of high-risk HPV infection (*P* = .001), which is consistent with Gilani and Mazzara’s report.^[29]^ However, Zhang et al^[30]^ analyzed HPV infection in 216 patients with pathological upgrading following conization and found that HPV infection is not associated with pathological upgrading. Hence, the effect of HPV infection on pathological upgrading in conization specimens remains to be further investigated.

AKbayir et al^[31]^ have reported that patients with postmenopausal period > 10 years are more likely to have pathological upgrading in conization specimens. In line with this finding, our result showed that patients with postmenopausal period ≥ 5 years were more likely to have pathological upgrading, which is consistent with Zhang et al’s study.^[14]^ These results suggest that the duration postmenopausal period can affect the incidence of pathological upgrading, which may be attributed to the fact that postmenopausal women pay less attention to regular screening, and delay treatment of abnormal conditions. In this study, EGI is a risk factor for pathological upgrading in perimenopausal women. The EGI suggests complicated pathological changes. At the same time, the common type of cervical transformation zone in perimenopausal women is type Ⅱ or type Ⅲ, which causes certain limitations in colposcopy biopsy. Zhu et al’s study^[32]^ suggested that in patients with glandular involvement, there were residual lesions on the conization specimens, which was 1 of the reasons for pathological upgrading.

Colposcopy-mediated multi-point biopsy is the main method for diagnosis of CIN. However, the cervical transformation zone of perimenopausal women is often type III, and the cervical canal lesions are often multicentric and cannot be detected. It has been shown that the accuracy of colposcopy-mediated multi-point biopsy is about 66% to 84% in the diagnosis of CIN.^[33]^ Therefore, colposcopy-mediated multi-point biopsy combined with ECC is increasingly common in cervical precancerous screening. Liu et al^[34]^ recommended routine ECC for women over 40 years of age with HPV16 infection. Hu et al^[35]^ found that in women with abnormal results of Thinprep Cytology Testing/HPV DNA testing but without abnormal colposcopic findings, a 4-quadrant random biopsy combined with ECC can detect high-grade squamous intraepithelial lesions or above lesions in 27.5% of all cases. Our result suggests that colposcopic cervical biopsy combined with ECC can increase the lesions detection rate, in turn reducing the risk of pathological upgrading. For perimenopausal women, routine ECC can be considered together with colposcopy biopsy to exclude cervical canal lesions. On the other hand, considering that the perimenopausal women have no fertility requirements, the cervical canal length can be measured by ultrasound before the operation and the surgical field can be fully exposed to ensure sufficient surgical scope, thus reducing the probability of postoperative pathological upgrading.

It should be pointed out that the current study was limited by its retrospective nature and small sample size. In addition, we did not collect cervical biopsy before conization for comparison. Therefore, a well-designed prospective trial should be conducted to validate the findings of this study.

## 5. Conclusions

In summary, this study suggested that pathological upgrading in conization specimens is not uncommon in Chinese CIN2/3 patients ≥ 50 years of age. For those with high-risk factors of pathological upgrading (postmenopausal period ≥ 5 years, EGI, and HPV 16/18 infection), endocervical scraping should be routinely performed concurrently along with colposcopy cervical biopsy to reduce the possibility of pathological upgrading.

## Author contributions

**Conceptualization:** Jumin Niu.

**Data curation:** Mingyu Jia, Chong Lan.

**Formal analysis:** Mingyu Jia, Chong Lan, Yin Liang.

**Funding acquisition:** Jumin Niu.

**Investigation:** Mingyu Jia, Chong Lan.

**Methodology:** Yin Liang.

**Project administration:** Jumin Niu.

**Resources:** Yin Liang.

**Software:** Yin Liang.

**Supervision:.** Jumin Niu.

**Validation:** Mingyu Jia.

**Visualization:** Mingyu Jia.

**Writing - original draft:** Mingyu Jia.

**Writing - review & editing:** Mingyu Jia.
